# Early age at menarche and the risk of gestational diabetes mellitus: a cohort study

**DOI:** 10.1007/s12020-024-03776-6

**Published:** 2024-03-18

**Authors:** Anastasia Angelopoulou, Kleoniki I. Athanasiadou, Maria Zairi, Evangelia Zapanti, Vasiliki Vasileiou, Stavroula A. Paschou, Eleni Anastasiou

**Affiliations:** 1 Department of Endocrinology and Diabetes Centre, Alexandra Hospital, Athens, Greece; 2 Endocrine Unit and Diabetes Centre, Department of Clinical Therapeutics, Alexandra Hospital, School of Medicine, National and Kapodistrian University of Athens, Athens, Greece

**Keywords:** GDM, Menarche, Obesity, Pregnancy

## Abstract

**Purpose:**

To evaluate whether there is an association between age at menarche (AAM) and the risk of gestational diabetes mellitus (GDM).

**Methods:**

A retrospective cohort study was conducted, including 5390 pregnant women who were screened for GDM at Alexandra Hospital in Athens, Greece over a 15-year period (2000–2014). Maternal age, pre-pregnancy body mass index (BMI), height, family history of type 2 diabetes mellitus, parity, educational and smoking status, and AAM were recorded. The results were expressed as odds ratios (OR) with a 95% confidence interval (95% CI).

**Results:**

Pregnant women with GDM experienced earlier menarche compared to normoglycemic women (12.9 ± 1.5 vs 13.1 ± 1.6, *p* < 0.001, respectively). The OR for a woman with AAM <12 years to develop GDM was 1.08 (95% CI 1.03–1.14), while the OR to be obese was 1.70 (95% CI 1.50–1.90). The multivariate logistic regression analysis showed that AAM is a risk factor for GDM. However, that effect was lost after adjusting for BMI.

**Conclusion:**

Early AAM may be associated with an increased risk of GDM. Therefore, it can be used to identify high-risk women and implement preconception interventions for GDM prevention. Future studies should be conducted to confirm these findings.

## Introduction

Gestational diabetes mellitus (GDM) is the most common metabolic complication of pregnancy. GDM’s prevalence is estimated at 9–14%, with a worldwide variation due to several socioenvironmental factors (maternal age, weight, nutrition, etc.) and the heterogenous diagnostic criteria that are used [1, 2]. GDM is related to various adverse outcomes for the mother and the offspring [3]. There are several known risk factors for GDM, such as advanced maternal age, maternal obesity, history of GDM in a previous pregnancy, and family history (FH) of diabetes mellitus. However, current research has revealed many more risk factors that had been underestimated in the past, while there are still more to be assessed [4–7]. Menarche is defined as the onset of menstruation and represents the female reproductive maturation. Normally, age at menarche (AAM) ranges between 9 and 16 years old [8]. It is influenced by genetic, hormonal, and environmental factors, including nutritional habits, physical activity, stressful situations, and endocrine-disrupting chemicals (EDCs) exposure [9, 10]. Early menarche is defined as the onset of menstruation before the age of 12 years old. It has been associated with an increased risk of type 2 diabetes mellitus (T2DM), obesity (body mass index, BMI >30 kg/m^2^), and cardiovascular disease [11]. However, the relationship between AAM and GDM remains inconsistent.

The onset of puberty is signaled by the stimulation of GnRH secretion exerted by the hypothalamic pulse generator. Several neuropeptides and hormones, including GABA, kisspeptin, and leptin, control the switch that triggers the hypothalamus [12]. According to the Nurses’ Health Study (NHS) and Nurses’ Health Study II (NHS II), every 1-year increase in menarcheal age is associated with a 6–10% reduction in T2DM risk [13]. The aim of the present study was to investigate the relationship between age at menarche and GDM and the possible mechanisms linking the two conditions. There is a limited number of relevant studies in the published literature. To our knowledge, this is the first study investigating that relationship in the Greek population.

## Materials and methods

The present study included 5390 pregnant women, who were screened for GDM at the Diabetes Center of Alexandra Hospital (University of Athens, Greece), which is a referral tertiary hospital, over a period of 15 years (2000–2014). All participants provided a written informed consent, so that their blinded data could be used in the present study. The Institutional Ethics Committee approved the conduction of the study.

All pregnant women underwent a 3 h, 100 g oral glucose tolerance test (OGTT) between the 24th–32nd gestational week. The OGTT was performed after a 12 h overnight fasting, while during the preceded 3 days all women had followed an unrestricted diet with ≥150 g carbohydrates/day. The Carpenter–Coustan diagnostic criteria were applied: (1) fasting glucose ≥95 mg/dl, (2) 1 h ≥180 mg/dl, (3) 2 h ≥155 mg/dl, (4) 3 h ≥140 mg/dl; the detection of two or more abnormal values led to GDM diagnosis. On the test day, age, pre-pregnancy BMI, height, FH of T2DM, parity, educational and smoking status, and age at menarche were recorded.

Venous blood was collected in BD Vacutainer® spray-coated K2EDTA tubes. Plasma glucose levels were determined by the glucose oxidase method (Integra/400 plus autoanalyzer, Roche Laboratory Systems). Glycated hemoglobin (HbA1c) was determined using high-pressure liquid chromatography (Menarini-Arkay ΗΑ-8160). The inter- and intra-assay coefficients of variation for all parameters were <5%.

### Statistical analysis

Data were expressed as mean ± standard deviation (SD) for continuous variables or as absolute numbers and percentages in parentheses for categorical variables. Logistic regression analyses were used to calculate the odds ratios (ORs) and 95% confidence intervals (95% CI). Differences between two continuous variables were determined using the t-test, while the chi^2^ test was used to test the difference between two categorical variables. In the multivariate logistic regression model, GDM was the dependent variable, and AAM <12 years, maternal age >25 years, BMI >30 kg/m^2^, and positive FH of T2DM were the independent variables. The level of significance was set at 5%. The statistical analyses were performed using the SPSS Statistics version 22.0, USA.

## Results

According to the OGTT results, 2452 were diagnosed with GDM, and 2938 women were considered normoglycemic (45.5 vs 54.5%). The great prevalence of GDM in our cohort is attributed to the high-risk pregnancies that are followed up at our tertiary referral hospital. The mean maternal age was 32.2 years old in the GDM group and 29.9 in the normoglycemic group. The mean AAM was 12.9 ± 1.5 and 13.1 ± 1.6 in women with and without GDM, respectively (p < 0.001). The summary of participant characteristics is presented in Table 1.

**Table 1:** Summary of participant characteristics

	GDM group	Normoglycemic group
*n* (%)	2452 (45.5%)	2938 (54.5%)
Maternal age (years)	32.2 ± 12.8	29.9 ± 11.6
BMI (kg/m^2^)	26.2 ± 4.8	25.5 ± 5.1
Age at menarche (years)	12.9 ± 1.5	13.1 ± 1.6
Family history of T2DM	958 (39.1%)	934 (31.8%)
Smoking	238 (9.7%)	305 (10.3%)

*BMI* body mass index, *T2DM* type 2 diabetes mellitus

**Fig. 1:**
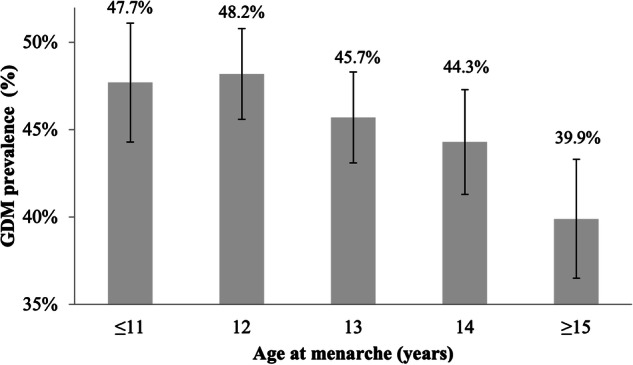
GDM prevalence according to age at menarche

The OR for women with AAM <12 years to develop GDM was 1.08 (95% CI 1.03–1.14). The association between AAM and the risk of GDM is presented at Fig. 1. Earlier age at menarche was associated with the positive FH of T2DM, as its prevalence was higher in women with AAM <12 years (39.1 vs 31.8%, *p* < 0.001).

**Fig. 2:**
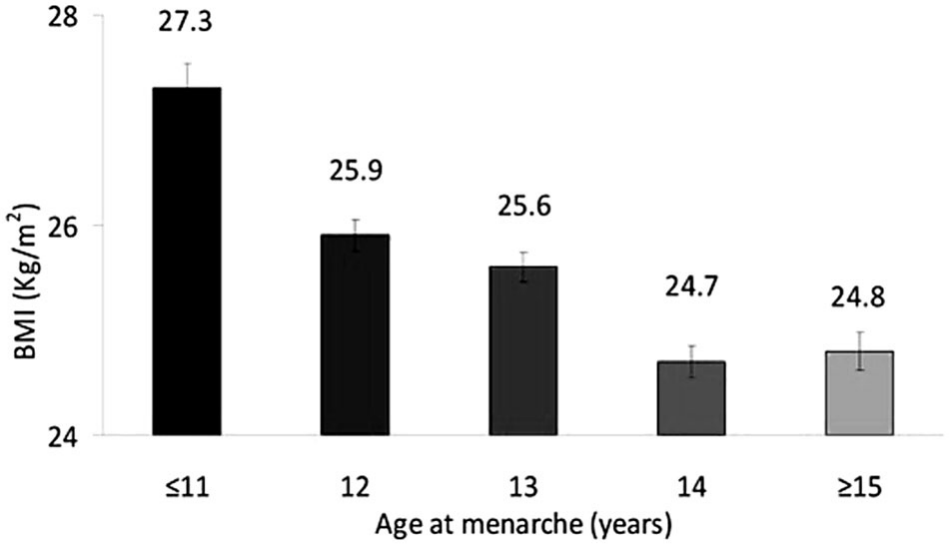
Association between maternal BMI and age at menarche

**Fig. 3:**
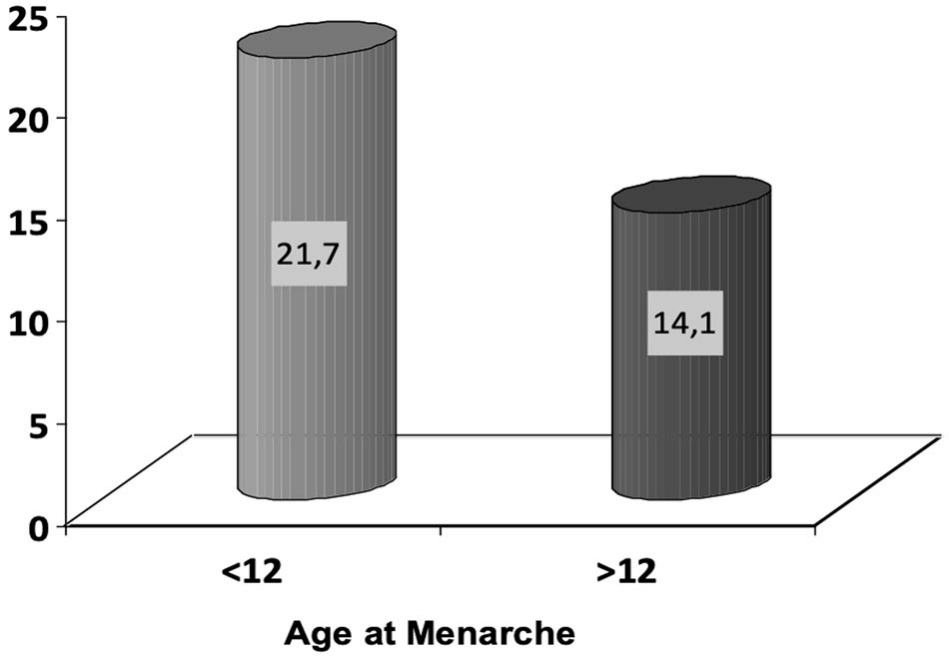
Prevalence of obesity according to the age at menarche

The multivariate logistic regression model concluded that AAM was an independent risk factor of GDM when adjusting for established risk factors, such as maternal age and FH of T2DM. However, the effect was lost with the addition of maternal BMI. The association between maternal BMI and AAM is presented at Fig. 2. Women with AAM <12 years had higher BMI compared to those with AAM >12 years (26.4 ± 5.9 vs 25.1 ± 5.0, *p* < 0.001). Furthermore, as it is shown in Fig. 3, the prevalence of obesity was significantly higher in women with AAM <12 years (21.7 vs 14.1%). The OR for a woman with AAM <12 years to be obese was 1.70 (95% CI 1.50–1.90).

## Discussion

Although there are several known risk factors for GDM, many more require investigation. The aim of the present study was to evaluate the association between age at menarche and GDM, as the published relevant studies are scarce. Our study concluded that early age at menarche is associated with an increased risk of GDM (OR 1.08, 95% CI 1.03–1.14), after adjusting for maternal age and FH of T2DM. The mean AAM in the present study participants was 12.9 ± 1.5. As the average AAM in the Greek population is 12 years it was used as a threshold to define early menarche [14]. Three systematic reviews and meta-analyses, published between 2018 and 2020, evaluated the relationship between AAM and GDM; they all support a strong association among them [15, 16]. A number of original studies presented below confirm our results, while one study published by Dishi et al. [17] did not detect an association between the two entities [17].

Chen et al. [18] proved that there is an association between earlier menarche and the risk of GDM (OR 1.13, 95% CI 0.97–1.31) by analyzing Nurses’ Health Study II data (n = 27,482). In line with our results, that effect was substantially attenuated when adjusting for prepregnancy BMI. The result was attributed to prepregnancy obesity, low levels of sex hormone-binding globulin (SHBG) and high levels of androgens and estrogens, which predispose to earlier menarche [18]. Shen et al. evaluated a nationally representative sample of 5914 women from the US National Health and Nutrition Examination Surveys (NHANES) and concluded that there is a 1.75-fold risk of developing GDM in women with earlier menarche. They stated that the decreased levels of adiponectin, which characterize pregnancy, may contribute to metabolic dysregulation, insulin resistance, and diabetes development. The alternative provided explanation supported that the hormonal status associated with early menarche exerts endocrine placental changes during pregnancy affecting glucose metabolism [19]. Moreover, Schoenaker et al. evaluated nationally representative data from 4749 women participating in the Australian Longitudinal Study on Women’s Health [20]. After adjusting for several confounders, they concluded that AAM ≤11 years was related to a 51% higher risk of GDM compared to women with AAM at 13 years. The proposed mechanisms included the mediation of childhood adiposity, the following predisposition to adulthood obesity, and the subsequent effect of estrogens and SHBG. Genetic predisposition to earlier menarche was also considered [21].

Li et al. [22] proved that early AAM is an independent risk factor for GDM (aOR 1.49, 95% CI 1.13–1.98, *n* = 6900) [22]. Researchers proposed that the pre-existing insulin resistance associated with earlier AAM becomes profound during pregnancy with the eventual development of GDM. Hormonal dysregulations (high estrogen, low SHBG levels) were also identified as possible causative factors [23]. Wang et al. [24] evaluated 70,041 women and showed that AAM between 8–12 years was associated with increased odds of GDM (OR 1.08, 95% CI 1.02–1.15), while the adjustment for prepregnancy BMI attenuated the result [24]. Ergin et al. revealed a 2.25-fold higher risk of GDM in women with AAM < 12 years, analyzing data from 373 women. They also proved that obesity increases the risk of GDM by three times (OR 3.07, 95% CI 1.57–5.95) [25]. The supported mechanism includes insulin resistance characterizing early AAM, which is further aggravated by the metabolic stress of pregnancy and the excess insulin demand. Lu et al. using data from the UK Biobank (n = 123,579), analyzed 113 genetic variants of estradiol, testosterone, and SHBG. Impressively, they showed that genetic predisposition to earlier AAM, expressed by single nucleotide polymorphisms (SNPs) in the aforementioned hormones, had a causal relationship with a higher risk of GDM [26].

Therefore, it seems that the relationship between age at menarche and GDM is primarily -but not exclusively- mediated by mechanisms related to maternal BMI. Obesity influences menarcheal age, as well as GDM and T2DM development, as these conditions share common pathogenetic pathways, mainly insulin resistance [27]. Prepregnancy obesity, childhood adiposity, hormonal changes in adiponectin, SHBG, estradiol, and testosterone, placental involvement, and genetic predisposition were the identified mechanisms linking the two conditions [28]. Obesity has a downstream effect on SHBG production. As a result, estrogen and androgen levels are higher in obese women, leading to earlier AAM and clinical features of hyperandrogenism [23, 29–32].

In line with the published literature, we concluded that maternal obesity increases the risk of GDM by almost two times (OR 1.70, 95% CI 1.50–1.90), as well as that the prevalence of obesity was much higher in women with earlier menarche. Two suggested mechanisms that may explain further our results involve leptin and the presence of overt or undiagnosed polycystic ovary syndrome (PCOS) [33]. Leptin is produced by the adipose cells and regulates appetite and satiety. Its serum concentration is strongly associated with body fat and directly reflects the body fat stores [34]. Leptin stimulates the hypothalamic-pituitary-gonadal (HPG) axis and affects menarche by interacting with the GnRH neurons via afferent interneurons [35]. The elevated fat stores increase the circulating leptin levels and gradually establish leptin resistance. Each 1 ng/ml increase in serum leptin leads to a 1-month decrease in age of menarche [36]. Conclusively, it seems that earlier menarche has an impact on maternal glucose metabolism through several mechanisms mediated by childhood and prepregnancy obesity, as well as by several hormonal fluctuations.

The current study has several strengths and certain limitations. The strengths of our study include the large sample size and the consistent GDM diagnostic criteria that were applied. Furthermore, the present study is the first one conducted in the Greek population using data from the largest national GDM registry and is one of the limited published studies evaluating the association between early AAM and GDM. Multivariate logistic regression analysis was performed, along with adjustment for several confounders, including maternal age at menarche, BMI, and FH of T2DM. Nevertheless, some limitations should be acknowledged. These are owing to the study’s retrospective nature and include the lack of information about maternal menstrual cycle characteristics (cycle length, regularity, and bleeding pattern) and the recall bias that may be present, as age at menarche was self-reported. Besides, another limitation is the use of the Carpenter–Coustan criteria, which may exclude some cases of GDM. However, the more sensitive and specific IADPSG criteria were recommended by the World Health Organisation in 2013 and were adopted by our Institution in 2016, while the study’s data refer to the 2000–2014 period. Finally, regarding the study’s high GDM prevalence (45.5%), the reproducibility of the results may be implemented only in other tertiary referral centers which share similar GDM prevalence in their registries.

In conclusion, early age at menarche, mediated by obesity and hormonal fluctuations, may be associated with an increased risk of GDM. The present study highlights the need to evaluate early age at menarche as a useful clinical information which can guide preconception interventions for GDM prevention. Future prospective studies are required to confirm our findings and expand our observations on the underlying mechanisms. The recall bias could be limited with the broader use of official registries incorporating the age at menarche and other relevant data from pediatric care, bridging the transition from childhood to the reproductive age.

## References

[1] H. Wang, N. Li, T. Chivese, M. Werfalli, H. Sun, L. Yuen et al. IDF diabetes atlas: estimation of global and regional gestational diabetes mellitus prevalence for 2021 by International Association of Diabetes in Pregnancy Study Group’s Criteria. Diabetes Res. Clin. Pract. **183**, 109050 (2022). 10.1016/j.diabres.2021.109050 34883186 10.1016/j.diabres.2021.109050

[2] E. Anastasiou, G. Farmakidis, A. Gerede, D.G. Goulis, E. Koukkou, A. Kourtis et al. Clinical practice guidelines on diabetes mellitus and pregnancy: ΙI. Gestational diabetes mellitus. Hormones **19**, 601–607 (2020). 10.1007/s42000-020-00193-y 32451981 10.1007/s42000-020-00193-y

[3] S.A. Paschou, E. Bletsa, M. Papazisi, N. Mili, F. Kanouta, G.N. Kassi, et al. Screening and management of major endocrinopathies during pregnancy: an update. Endocrine 10–19 (2022). 10.1007/s12020-022-03237-y. 10.1007/s12020-022-03237-y PMC10060311 36327019

[4] D. Eleftheriou, K.I. Athanasiadou, E. Sifnaios, E. Vagiakis, P. Katsaounou, T. Psaltopoulou, et al. Sleep disorders during pregnancy: an underestimated risk factor for gestational diabetes mellitus. Endocrine (2023). 10.1007/s12020-023-03537-x. 10.1007/s12020-023-03537-x PMC10805805 37740834

[5] K.I. Athanasiadou, S.A. Paschou, E. Papakonstantinou, V. Vasileiou, F. Kanouta, P. Kazakou, et al. Smoking during pregnancy and gestational diabetes mellitus: a systematic review and meta-analysis. Endocrine (2023). 10.1007/s12020-023-03423-6. 10.1007/s12020-023-03423-6 PMC10543648 37347387

[6] P. Konstantakou, S.A. Paschou, I. Patinioti, E. Vogiatzi, V. Sarantopoulou, E. Anastasiou, The effect of smoking on the risk of gestational diabetes mellitus and the OGTT profile during pregnancy. Diabetes Res. Clin. Pract. **158**, 107901 (2019). 10.1016/j.diabres.2019.107901 31669407 10.1016/j.diabres.2019.107901

[7] V. Vasileiou, E. Kyratzoglou, S.A. Paschou, M. Kyprianou, E. Anastasiou, The impact of environmental temperature on the diagnosis of gestational diabetes mellitus. Eur. J. Endocrinol. **178**, 209–214 (2018). 10.1530/EJE-17-0730 29363527 10.1530/EJE-17-0730

[8] I. Culpin, J. Heron, R. Araya, R. Melotti, G. Lewis, C. Joinson, Father absence and timing of menarche in adolescent girls from a UK cohort: The mediating role of maternal depression and major financial problems. J. Adolesc. **37**, 291–301 (2014). 10.1016/j.adolescence.2014.02.003 24636689 10.1016/j.adolescence.2014.02.003

[9] J.R. Roy, S. Chakraborty, T.R. Chakraborty, Estrogen-like endocrine disrupting chemicals affecting puberty in humans - a review. Med. Sci. Monit. **15**, 137–145 (2009). 19478717

[10] K.L. Land, F.G. Miller, A.C. Fugate, P.R. Hannon, The effects of endocrine-disrupting chemicals on ovarian- and ovulation-related fertility outcomes. Mol. Reprod. Dev. **89**, 608–631 (2022). 10.1002/mrd.23652 36580349 10.1002/mrd.23652 PMC10100123

[11] N.T. Mueller, B.B. Duncan, S.M. Barreto, D. Chor, M. Bessel, E.M.L. Aquino et al. Earlier age at menarche is associated with higher diabetes risk and cardiometabolic disease risk factors in Brazilian adults: Brazilian Longitudinal Study of Adult Health (ELSA-Brasil). Cardiovasc. Diabetol. **13**, 1–8 (2014). 10.1186/1475-2840-13-22 24438044 10.1186/1475-2840-13-22 PMC3899384

[12] M. Amstalden, B.R.C. Alves, S. Liu, R.C. Cardoso, G.L. Williams, Neuroendocrine pathways mediating nutritional acceleration of puberty: Insights from ruminant models. Front. Endocrinol. **2**, 1–7 (2011). 10.3389/fendo.2011.00109 10.3389/fendo.2011.00109 PMC3356117 22654842

[13] C. He, C. Zhang, D.J. Hunter, S.E. Hankinson, G.M. Buck Louis, M.L. Hediger et al. Age at menarche and risk of type 2 diabetes: Results from 2 large prospective cohort studies. Am. J. Epidemiol. **171**, 334–344 (2010). 10.1093/aje/kwp372 20026580 10.1093/aje/kwp372 PMC2842205

[14] O. Karapanou, A. Papadimitriou, Determinants of menarche. Reprod. Biol. Endocrinol. **8**, 1–8 (2010). 10.1186/1477-7827-8-115 20920296 10.1186/1477-7827-8-115 PMC2958977

[15] Y. Ren, H. Zou, D. Zhang, C. Han, D. Hu, Relationship between age at menarche and risk of glucose metabolism disorder: a systematic review and dose-response meta-analysis. Menopause **27**, 818–826 (2020). 10.1097/GME.0000000000001529 32217891 10.1097/GME.0000000000001529

[16] X. Sun, L. Yang, J. Pan, H. Yang, Y. Wu, Z. Chen et al. Age at menarche and the risk of gestational diabetes mellitus: a systematic review and meta-analysis. Endocrine **61**, 204–209 (2018). 10.1007/s12020-018-1581-9 29556913 10.1007/s12020-018-1581-9

[17] M. Dishi, D.A. Enquobahrie, D.F. Abetew, C. Qiu, C.B. Rudra, M.A. Williams, Age at menarche, menstrual cycle characteristics and risk of gestational diabetes. Diabetes Res. Clin. Pract. **93**, 437–442 (2011). 10.1016/j.diabres.2011.07.001 21816498 10.1016/j.diabres.2011.07.001

[18] L. Chen, S. Li, C. He, Y. Zhu, G.M. Buck Louis, E. Yeung et al. Age at menarche and risk of gestational diabetes mellitus: a prospective cohort study among 27,482 women. Diabetes Care **39**, 469–471 (2016). 10.2337/dc15-2011 26813668 10.2337/dc15-2011 PMC4764040

[19] Y. Shen, H. Hu, B. D. Taylor, H. Kan, X. Xu, Early menarche and gestational diabetes mellitus at first live birth. Matern. Child Health J. **21**, 593–598 (2017). 10.1007/s10995-016-2143-5 27456304 10.1007/s10995-016-2143-5

[20] D.A.J.M. Schoenaker, G.D. Mishra, Association between age at menarche and gestational diabetes mellitus the Australian longitudinal study on women’s health. Am. J. Epidemiol. **185**, 554–561 (2017). 10.1093/aje/kww201 28338812 10.1093/aje/kww201

[21] D.H. Morris, M.E. Jones, M.J. Schoemaker, A. Ashworth, A.J. Swerdlow, Familial concordance for age at menarche: analyses from the Breakthrough Generations study. Paediatr. Perinat. Epidemiol. **25**, 306–311 (2011). 10.1111/j.1365-3016.2010.01183.x 21470270 10.1111/j.1365-3016.2010.01183.x

[22] H. Li, L. Shen, L. Song, B. Liu, X. Zheng, S. Xu et al. Early age at menarche and gestational diabetes mellitus risk: results from the Healthy Baby Cohort study. Diabetes Metab. **43**, 248–252 (2017). 10.1016/j.diabet.2017.01.002 28161369 10.1016/j.diabet.2017.01.002

[23] M.M. Hedderson, F. Xu, J.A. Darbinian, C.P. Quesenberry, S. Sridhar, C. Kim et al. Prepregnancy SHBG concentrations and risk for subsequently developing gestational diabetes mellitus. Diabetes Care **37**, 1296–1303 (2014). 10.2337/dc13-1965 24561392 10.2337/dc13-1965 PMC3994937

[24] L. Wang, B. Yan, X. Shi, H. Song, W. Su, B. Huang et al. Age at menarche and risk of gestational diabetes mellitus: a population-based study in Xiamen, China. BMC Pregnancy Childbirth **19**, 1–7 (2019). 10.1186/s12884-019-2287-6 31023245 10.1186/s12884-019-2287-6 PMC6482560

[25] A. Ergin, Ü. Türkay, S. Özdemir, A. Taşkın, H. Terzi, M. Özsürmeli, Age at menarche: risk factor for gestational diabetes. J. Obstet. Gynaecol. **42**, 680–686 (2022). 10.1080/01443615.2021.1929116 34415226 10.1080/01443615.2021.1929116

[26] L. Lu, B. Wan, M. Sun, Mendelian randomization identifies age at menarche as an independent causal effect factor for gestational diabetes mellitus. Diabetes Obes. Metab. **25**, 248–260 (2023). 10.1111/dom.14869 36106372 10.1111/dom.14869

[27] D.S. Freedman, L. Kettel Khan, M.K. Serdula, W.H. Dietz, S.R. Srinivasan, G.S. Berenson, The relation of menarcheal age to obesity in childhood and adulthood: the Bogalusa Heart study. BMC Pediatr. **3**, 1–9 (2003). 10.1186/1471-2431-3-3 12723990 10.1186/1471-2431-3-3 PMC156622

[28] S. Ramachandran, G.I. Hackett, R.C. Strange, Sex hormone binding globulin: a review of its interactions with testosterone and age, and its impact on mortality in men with type 2 diabetes. Sex. Med. Rev. **7**, 669–678 (2019). 10.1016/j.sxmr.2019.06.006 31447413 10.1016/j.sxmr.2019.06.006

[29] D. Apter, M. Reinilä, R. Vihko, Some endocrine characteristics of early menarche, a risk factor for breast cancer, are preserved into adulthood. Int. J. Cancer **44**, 783–787 (1989). 10.1002/ijc.2910440506 2511157 10.1002/ijc.2910440506

[30] M.A. Tawfeek, E.M. Alfadhli, A.M. Alayoubi, H.A. El-Beshbishy, F.A. Habib, Sex hormone binding globulin as a valuable biochemical marker in predicting gestational diabetes mellitus. BMC Womens Health **17**, 3–7 (2017). 10.1186/s12905-017-0373-3 28279160 10.1186/s12905-017-0373-3 PMC5345161

[31] A. Thankamony, K.K. Ong, M.L. Ahmed, A.R. Ness, J.M.P. Holly, D.B. Dunger, Higher levels of IGF-I and adrenal androgens at age 8 years are associated with earlier age at menarche in girls. J. Clin. Endocrinol. Metab. **97**, 786–790 (2012). 10.1210/jc.2011-3261 10.1210/jc.2011-3261 PMC3444968 22419724

[32] E.L. Ding, Y. Song, V.S. Malik, S. Liu, Sex differences of endogenous sex hormones and risk of type 2 diabetes. Jama **295**, 1288 (2006). 10.1001/jama.295.11.1288 16537739 10.1001/jama.295.11.1288

[33] M. Sawada, H. Masuyama, K. Hayata, Y. Kamada, K. Nakamura, Y. Hiramatsu, Pregnancy complications and glucose intolerance in women with polycystic ovary syndrome. Endocr. J. **62**, 1017–1023 (2015). 10.1507/endocrj.EJ15-0364 26370557 10.1507/endocrj.EJ15-0364

[34] R.V. Considine, M.K. Sinha, M.L. Heiman, A. Kriauciunas, T.W. Stephens, M.R. Nyce et al. Serum immunoreactive-leptin concentrations in normal-weight and obese humans. N. Engl. J. Med. **334**, 292–295 (1996). 10.1056/NEJM199602013340503 8532024 10.1056/NEJM199602013340503

[35] J.H. Quennell, A.C. Mulligan, A. Tups, X. Liu, S.J. Phipps, C.J. Kemp et al. Leptin indirectly regulates gonadotropin-releasing hormone neuronal function. Endocrinology **150**, 2805–2812 (2009). 10.1210/en.2008-1693 19179437 10.1210/en.2008-1693 PMC2732287

[36] V. Matkovic, J.Z. Ilich, M. Skugor, N.E. Badenhop, P. Goel, A. Clairmont et al. Leptin is inversely related to age at menarche in human females. J. Clin. Endocrinol. Metab. **82**, 3239–3245 (1997). 10.1210/jc.82.10.3239 9329346 10.1210/jc.82.10.3239

